# Predicting Cognitive Decline: A Comprehensive Study On relative Brain Age, Cognitive Reserve, And Longitudinal Changes

**DOI:** 10.1002/alz.093733

**Published:** 2025-01-09

**Authors:** Mahboubeh Motaghi, Olivier Potvin, Valérie Turcotte, Iman Beheshti, Simon Duchesne

**Affiliations:** ^1^ Laval University, Quebec City, QC Canada; ^2^ Centre de recherche de l'Institut Universitaire de Cardiologie et de Pneumologie de Québec, Québec, QC Canada; ^3^ Université Laval, Québec, QC Canada; ^4^ University of Manitoba, Winnipeg, MB Canada; ^5^ Centre de recherche de l'Institut Universitaire de Cardiologie et de Pneumologie de Québec, Québec city, QC Canada

## Abstract

**Background:**

Brain age is a metric that can be determined through anatomical measurements obtained from MRI scans. Relative brain age (RBA) reflects the difference in brain morphometry for an individual relative to a comparison group at a similar chronological age. Increased RBA was found related to poor physical health, neurodegeneration, increase RBA, cognitive decline, and mortality risk. The concept of cognitive reserve (CR), operationalized by merging education, complexity of occupation, and verbal IQ, explains on the other hand how individuals seem able to tolerate the impact of age‐related and/or neurodegenerative changes. This study aimed at exploring the relationship between RBA, CR, and cognitive performance.

**Method:**

We assessed cognitively healthy and MCI individuals from the ADNI study on four cognitive domains (verbal episodic memory; language and semantic memory; attention; executive functions) at each follow‐up visit up to 5 years. First, we employed robust statistical models, including Generalized Linear Models(GLM), to investigate the association between baseline variables and cognitive performance changes over different follow‐up intervals. Secondly, we used support vector regression models to predict cognitive performance over multiple follow‐up periods. We employed 10‐fold cross‐validation to assess the predictive accuracy of the model. Finally, we used GLM to assess the link between RBA and CR with the speed of cognitive changes during a 36‐month period.

**Result:**

Our findings reveal that in MCI group, RBA significantly contributes to the variance in verbal episodic memory changes across various time intervals. To predict cognitive performance, our support vector regression model demonstrated robust performance, yielding low mean absolute errors for episodic memory (0.305 to 0.523), attention (0.392 to 0.661), language and semantic memory (0.369 to 0.537), and executive function (0.386 to 0.521) across diverse follow‐up periods. RBA was further associated with a slower decline in verbal episodic memory, attention, language and semantic memory. These findings underscore the significant predictive role of RBA in estimating the speed of decline in cognitive performance within the MCI group.

**Conclusion:**

We have shown how RBA interacts with CR, in shaping cognitive performance and aging trajectories. Our findings underscore the pivotal role of RBA in influencing future cognitive decline, offering valuable insights for early clinical detection.

